# Elastic Stable Intramedullary Nailing for Pediatric Humeral Shaft Fractures Under Ultrasonographic Guidance: A Retrospective Study

**DOI:** 10.3389/fped.2021.806100

**Published:** 2022-01-26

**Authors:** Jun Li, Jun Wu, Yuan Zhang, Pan Gou, Xiang Li, Mingyan Shi, Man Zhang, Peikang Wang, Xing Liu

**Affiliations:** Department of Orthopedic, Ministry of Education Key Laboratory of Child Development and Disorders, National Clinical Research Center for Child Health and Disorders, China International Science and Technology Cooperation Base of Child Development and Critical Disorders Chongqing Key Laboratory of Pediatrics, Children's Hospital of Chongqing Medical University, Chongqing, China

**Keywords:** ultrasonography, humeral shaft fracture, close reduction, pediatric fracture, radiation exposure

## Abstract

**Objective:**

Fixation with an elastic stable intramedullary nail (ESIN) is a widely used technique for the treatment of humeral shaft fractures. Ultrasonography (US) is used as an auxiliary tool and alternative to radiography during surgery to reduce radiation damage, but whether it is effective in pediatric patients is not known. In this study we evaluated the utility of US in the treatment of pediatric humeral shaft fractures by closed reduction and fixation with an ESIN.

**Methods:**

Children who were admitted to our hospital with humeral shaft fractures were retrospectively examined from January 2016 to March 2019. The patients were divided into 2 groups, US (treated by US-guided closed reduction and ESIN fixation) and non-US (treated with the same technique but with intraoperative radiography instead of US). The postoperative functional recovery of the 2 groups was compared.

**Results:**

The study population comprised 28 boys and 17 girls (age range: 4–16 years) with humeral shaft fractures. US significantly reduced radiation exposure during the operation (*P* = 0.008), and intraoperative US facilitated the detection of nerve and vascular injury and aided surgical planning. There was no difference between the 2 groups in postoperative recovery based on the Constant–Murley shoulder score (CMS).

**Conclusions:**

These results demonstrate that US-guided closed reduction and ESIN fixation for humeral shaft fractures can limit radiation exposure and help doctors to determine the optimal surgical strategy to avoid radial nerve injury.

## Introduction

Humeral shaft fractures in children are exceedingly rare, accounting for 0.4–3% of all pediatric fractures with higher incidences in children younger than 3 years or older than 10 years (1–4). A conservative treatment approach that includes functional bracing, skin traction, and casts is used in these patients, which is associated with a good prognosis (5); however, there are also certain shortcomings such as skin damage at the traction site, long bed rest and hospitalization for patients with traction, and potential displacement of fractures (6).

Closed reduction and fixation with an elastic stable intramedullary nail (ESIN) is an excellent choice for the treatment of pediatric humeral shaft fractures, especially for older children. The advantages of this approach include minimal invasiveness, rigid fixation of fracture sites, a short hospitalization, and early postoperative functional recovery, although complications such as skin lesions, surgical site infection, and iatrogenic fracture can arise (7, 8). Additionally, both patients and doctors are exposed to high-dose radiation from X-rays used to visualize a closed fracture site with traditional surgical methods (9, 10).

Ultrasonic technology has the advantages of portability, non-invasiveness, and painlessness, and it is now used extensively for the diagnosis and treatment of fractures in children (11, 12). Ultrasonography (US) is superior to radiography for the precise assessment of radial nerve injury in patients with humeral shaft fractures (13). In our clinical experience, US-guided closed reduction and ESIN fixation of pediatric humeral shaft fractures is feasible. The aim of this retrospective study was to evaluate the utility of US for the surgical treatment of ESIN-treated humeral shaft fractures.

## Materials and Methods

### Setting

Children who were admitted to our hospital with humeral shaft fractures from January 2016 to March 2019 were included in this analysis. The inclusion criteria were as follows: (1) <16 years of age; and (2) humeral shaft fracture treated with an ESIN. Exclusion criteria were as follows: (1) multiple fractures, open fractures, or pathologic or comminuted fractures; (2) failure of closed reduction; (3) oblique fractures requiring fixation with a Kirschner wire; (4) incomplete follow-up; and (5) fractures with neurovascular involvement. We started using US to guide closed reduction treatment of humeral shaft fractures with ESIN in February 2018. Patients who were admitted between February 2018 and March 2019 constituted the US group, whereas those who were admitted from January 2016 to February 2018 were the non-US group (without US). All patients underwent routine postoperative follow-up for at least 12 months and were given exercises for functional recovery, which was assessed with the Constant–Murley shoulder (CMS) score (14) at the last follow-up.

Consent for study participation was obtained from the guardian of each patient and the study protocol was approved by the ethics committee of our hospital. Dates in the study were obtained from hospital records.

### Surgical Procedure

US was performed using a model CX50 color ultrasonic diagnostic apparatus (Philips, Amsterdam, Netherlands) with an L3-12 high-frequency linear probe and probe frequency of 5 MHz. Bedside C-arm fluoroscopy was performed with a model uMC 560i instrument (United Imaging, Shanghai, China). All surgeries were performed by 3 pediatric orthopedic surgeons who were experienced in the ESIN technique and US, and the same ESIN configuration was used in all patients.

**Figure 1:**
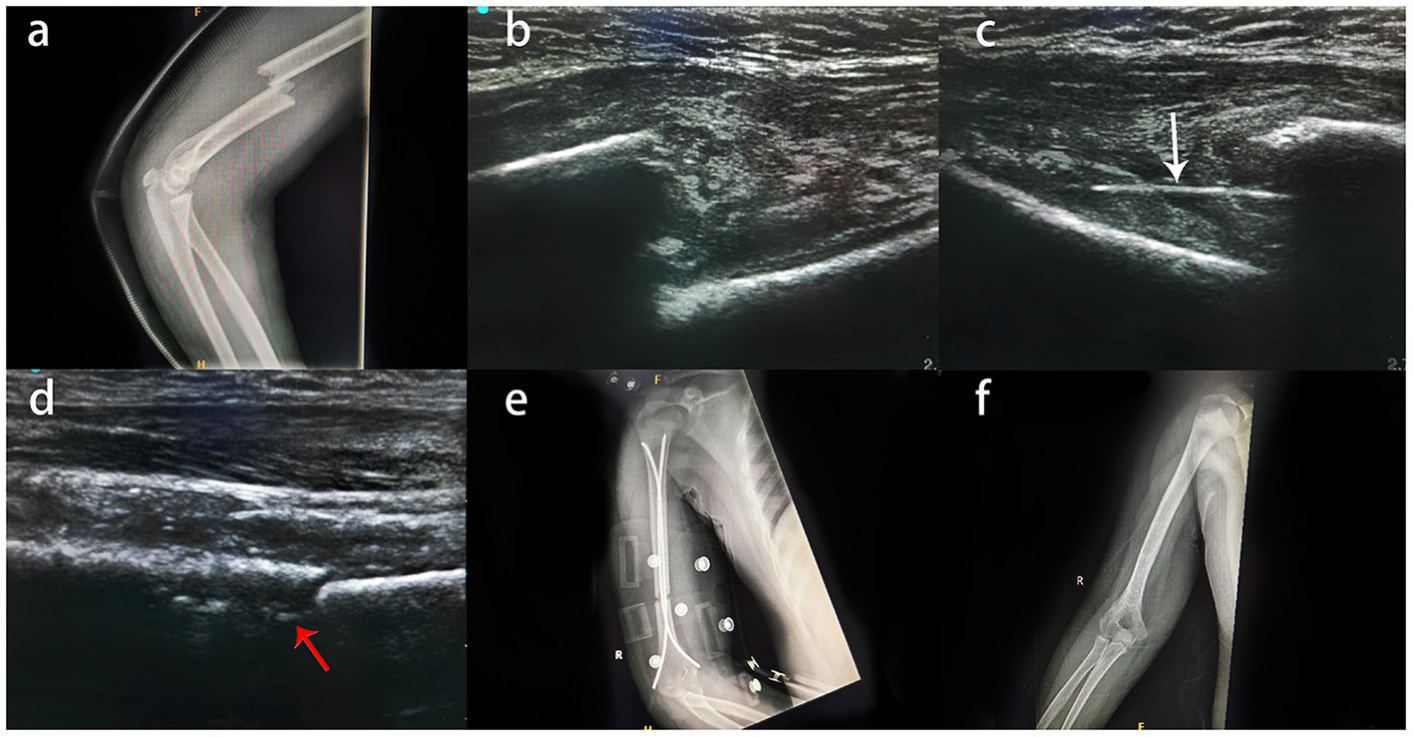
Typical case of an 11-year-old boy with right humeral shaft fracture treated by US-guided closed reduction and ESIN fixation. **(A)** X-ray examination of the child after injury revealed a middle humeral shaft fracture. **(B)** Intraoperative US showed that there was no soft tissue or nerve incarceration at the fracture site, and closed reduction was possible. **(C)** ESIN just past the fracture (white arrow). **(D)** ESIN in the bone marrow cavity after closed reduction (red arrows); it was not detectable in every patient by US. **(E)** Reexamination by radiography on the first day after the operation. **(F)** Good fracture healing was observed by X-ray at the 12-month follow-up.

Routine preoperative preparation was performed using a sterile endoscope cover-wrapped probe with iodophor as the ultrasonic couplant. The fracture was examined by US to determine whether there was soft tissue or nerve incarceration and whether closed reduction was feasible. A 1–1.5-cm skin incision was made at the lateral epiphysis of the distal humerus, and the soft tissue was separated. The distal humerus was perforated with a bone cone (Johnson & Johnson, New Brunswick, NJ) while avoiding damage to the epiphyseal plates. A pre-bent ESIN (Johnson & Johnson) of the proper diameter was slowly inserted into the hole, using US to monitor whether the ESIN exited at the fracture site. If the ESIN was difficult to insert or was not observed at the broken end of the fracture, the C-arm X-ray was used to determine its position in the marrow cavity. The bone cortex and ESIN are bright and hyperechoic on US images and are thus easily identified. When the ESIN was detected at the fracture site, it was slowly retracted into the marrow cavity. Closed reduction of the fracture appeared as an approximately straight line of cortical echo by US. The ESIN was subsequently reinserted past the fracture site. US was used to examine the fracture site from all directions to ensure that the ESIN did not protrude from the bone marrow cavity. Sometimes the ESIN in the marrow cavity was also observable at a specific location by US. After the ESIN was advanced to the appropriate site, its position was confirmed by radiography. The operation was repeated at the epiphysis of the medial condyle of the distal humerus, with care taken to avoid damaging the ulnar nerve (Figure 1). After surgery, the limb was immobilized with a functional brace or plaster.

### Follow-Up

All patients underwent X-ray examination on the first day after surgery and were discharged for follow-up in an outpatient clinic on Day 2 if there were no exceptional circumstances. The incision was verified 7 days after surgery and X-ray examination was performed at 3 and 6–8 weeks and 4, 6, and 12 months. Functional exercises were started 6–8 weeks after the surgery by the patients with the aid of family members who were given instructions on the exercises. Anteroposterior and lateral radiographs of the humerus were reviewed at each visit to evaluate callus formation at the fracture site and identify complications such as secondary displacement, delayed union, nonunion, or malunion. All patients were followed up for at least 12 months and shoulder function was evaluated based on CMS at the last follow-up.

### Statistical Analysis

SPSS v25 (IBM, Armonk, NY) was used for statistical analyses. Differences between categorical variables were evaluated with Pearson's χ2 test. The *P* value threshold for significance was set at 0.05.

## Results

### Characteristics of the Study Population

The study population comprised 28 boys and 17 girls; 24 children were assigned to the US group and 21 to the non-US group. There was no significant difference in sex ratio, age, or fracture location between the 2 groups (Table 1). Both groups had a hospitalization time of about 5 days, which was much shorter than for patients treated by skin traction. The average operation time was slightly shorter in the US group than in the non-US group, but the difference was not statistically significant. The average number of radiographs during the operation was 4.9 ± 1.92 (times) for the US group, which was fewer than for the non-US group (*P* = 0.008). Accordingly, radiation exposure was lower for doctors and children in the US group than in the non-US group.

**Table 1:** Demographic characteristics and clinical data of the patients.

	**US group**	**Non-US group**	**P**
Sex			
Male	15	13	0.967
Female	9	8	
Mean age (years)	9.8 ± 2.78	9.5 ± 2.94	0.782
Fracture location			
Proximal third	6	6	0.493
Middle third	15	11	
Distal third	3	4	
Surgery time (min)	46.0 ± 5.84	54.9 ± 6.70	0.522
Times of X-radiographs	4.9 ± 1.92	20.7 ± 3.45	0.008
Radiation of the X-rays (mGy)	1.36 ± 0.54	5.79 ± 0.97	0.008
Average time to surgery (days)	2.3 ± 0.74	2.5 ± 0.81	0.645
Length of hospital stay (days)	4.6 ± 1.18	4.8 ± 1.17	0.979
Average follow-up (months)	14.8 ± 3.53	16.6 ± 4.99	0.112
CMS			
Excellent (>90)	18	17	0.729
Good (81–90)	5	3	
Fair (61–80)	1	1	
Poor (<60)	0	0	
Complications			
Pin site infection	1	1	1
Radial nerve injury	0	0	
Bone nonunion	0	0	
Patient satisfaction (0–100)	96.0 ± 5.78	96.7 ± 5.99	0.862

*Data are shown as mean ± standard deviation unless indicated otherwise*.

*CMS, Constant–Murley shoulder; US, ultrasonography*.

### Clinical Outcomes

**Figure 2:**
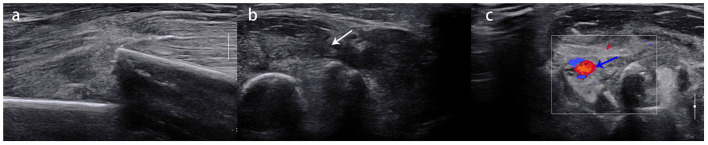
**(A)** A 9-year-old girl with right humeral shaft fracture. **(B)** Swelling of the radial nerve was observed (white arrow) but there was no rupture or entrapment. **(C)** Good blood supply to brachial artery (blue arrow) was observed at the fracture site.

All patients were followed up for at least 12 months, and limb function was evaluated with the CMS at the last follow-up. The rate of excellent or good CMS scores in the US group was 95.8 vs. 95.2% in the non-US group; there was no significant difference between groups. One of the most common side effects of a humeral shaft fracture is radial nerve damage (13). Because of the pain caused by the fracture, patients did not cooperate with the physical examination and therefore, the degree of radial nerve injury could not be judged solely by physical signs. By US, we could clearly determine whether the radial nerve was ruptured and compressed and evaluate whether open surgery was needed (Figure 2). There were 2 children in the US group and 1 in the non-US group with signs of radial nerve injury at admission, but all symptoms of injury in all children disappeared during follow-up. There was no significant difference in hospital satisfaction between the 2 groups, but according to our clinical experience, the cooperation of patients' family members improved when they were informed that radiation exposure during surgery was significantly reduced.

## Discussion

Closed reduction with ESIN fixation of humeral shaft fractures has the advantages of being minimally invasive, providing rigid fixation, and shortening the hospital stay, and is accepted by an increasing number of patients (7, 8, 15). As the fracture site cannot be viewed directly during closed reduction, it is necessary to repeatedly check radiographs during the operation to determine the extent of fracture reduction and position of the ESIN. However, this increases radiation exposure time, which in turn increases the risk of cancers such as thyroid, breast, brain, and skin cancers as well as leukemia, especially in children. Radiation-related cancer risk is greater in younger people and lasts a lifetime (16–18). Thus, examination methods that minimize damage during diagnosis and treatment are desired. The results of this study showed that the use of US reduced the intraoperative radiation exposure of our pediatric patients, and the follow-up results were also satisfactory. Minimally invasive surgery preserves soft tissue and decreases the risk of complications but is associated with greater radiation exposure than open procedures (19). The improved surgical method used in this study could substantially reduce radiation injury and maximize the clinical benefit to children.

Because of its multiplanar real-time imaging capabilities, cost-effectiveness, mobility, and lack of radiation exposure, US is increasingly employed in musculoskeletal system examinations (20, 21). In a study of 201 children with forearm fractures, US had a sensitivity and specificity of 99.5% in identifying fractures (22). Our institution also uses US in the treatment of displaced radial neck fractures; US guidance can reduce X-ray exposure and the risk of posterior interosseous nerve damage (12). We demonstrated that intraoperative US has incomparable advantages over radiography in soft tissue imaging as it can reveal whether the fracture is causing serious soft tissue injury or compression or cutting off the blood supply, which slows fracture healing (23). Additionally, it allows better design of the surgical plan and evaluation of the feasibility of closed reduction.

The radial nerve is one of the most susceptible nerves in a humeral shaft fracture (3); unless it is entrapped or ruptured, in most cases injuries will heal with conservative care (13). In this study, humeral shaft fractures were treated by closed reduction. Because the radial nerve could not be directly viewed, it is possible that it was trapped to the broken end of the fracture, which could aggravate an injury or even lead to sequelae that necessitate open surgery (24). When assessing nerve injury, US has a significant advantage over radiography and can be used to assess radial nerve damage caused by humeral shaft fractures in pediatric patients and predict prognosis (13). By intraoperative US we were able to determine whether the radial nerve was compressed at the fracture end or ruptured and decide whether to perform closed reduction, thereby minimizing the risk of nerve injury associated with this procedure.

Although there are many advantages to using US in the treatment of humeral shaft fractures by closed reduction, ultrasound cannot penetrate the cortical bone to enable visualization of the location of the ESIN. X-ray examination after the nails are implanted or during closed reduction is difficult, and open reduction may be necessary. Extending the operative time and thus prolonging anesthesia to reduce radiation exposure or surgical trauma is not desirable. Thus, US cannot completely replace the role of radiography in surgery.

In the present study, we did not observe that US conferred obvious advantages in terms of avoiding radial nerve injury, possibly because of the small sample size. The subjective factor of patient satisfaction could not be clearly evaluated but in our clinical experience, we feel that the degree of satisfaction among patients' families has improved. There was no significant difference in operative time between the 2 groups. In the early stage of this technique, the operative time was slightly longer than with conventional surgery, but with the increasing proficiency of the surgeons, this gradually improved. The C-arm position should be adjusted, and the surgeons must wait for the anesthesiologist and nurse to leave the operating room before performing the X-ray; however, for US it is only necessary to place the US probe on the skin to obtain images, which reduces operative time.

The main shortcoming of this study was the small sample size and the fact that patients were only followed for a brief period. There was also unavoidable sampling bias in the selection of the cohort.

## Conclusions

The results of this study show that intraoperative US cannot completely replace the role of radiography in humeral fracture surgery but can substantially reduce radiation exposure in pediatric patients and doctors. The detection of radial nerve and soft tissue injury caused by humeral shaft fracture by US can help doctors plan the appropriate surgical method to avoid aggravating radial nerve injury. In summary, US-guided closed reduction and ESIN fixation for humeral shaft fractures is a good surgical approach in pediatric patients.

## Data Availability Statement

The original contributions presented in the study are included in the article/supplementary material, further inquiries can be directed to the corresponding author/s.

## Author Contributions

JL and XLiu wrote the original paper and reviewed, revised it, and the project was conceptualized and designed. JW, YZ, PG, XLi, MZ, PW, and MS acquired the information, performed a preliminary analysis, and updated the paper. All authors agree to be accountable for all parts of the work and accept the final submission.

## Funding

This study was supported by Chongqing Science and Technology Commission Basic and Frontier Exploration General Project (No. csct2018jcyjA0259), the Key Project of Chongqing Health Planning Commission of Research Fund (No. 2019ZDXM047), and Yuzhong Science and Technology Commission Basic and Frontier Exploration General Project (No. 20180115).

## Conflict of Interest

The authors declare that the research was conducted in the absence of any commercial or financial relationships that could be construed as a potential conflict of interest.

## Publisher's Note

All claims expressed in this article are solely those of the authors and do not necessarily represent those of their affiliated organizations, or those of the publisher, the editors and the reviewers. Any product that may be evaluated in this article, or claim that may be made by its manufacturer, is not guaranteed or endorsed by the publisher.
